# Supplementary material for: Characterization of the clade 4 non-toxigenic C. difficile isolate L-NTCD03 carrying the cfr(B) gene
Source: FEMS Microbes. 2026 Feb 23;7:xtag010. doi: 10.1093/femsmc/xtag010 (PMC12955701; doi:10.1093/femsmc/xtag010)
Supplement: xtag010_Supplemental_Files [file xtag010_supplemental_files.zip › Nibbering_LNTCD03_feb2026_Supplemental.doc]

**Characterization of the clade 4 non-toxigenic *C. difficile* isolate L-NTCD03 carrying the *cfr*(B) gene**

Britt Nibbering^1^, Sam Nooij^1,2^, Céline Harmanus^2^, Ingrid M.J.G. Sanders^2^, Inez M. Miedema^2^, Quinten R. Ducarmon^1,2^, Rolf H.A.M. Vossen^3^, Susan L. Kloet^3^, Colleen K. Ardis^4^, Robert A. Britton^4^, Farnaz Yousefi^5^, Jenna Bayne^6,7^, Chandru Charavaryamath^8,9^, Andy Law^10^, Morgan L. Murphy^10,11^, Brett Sponseller^12,13^, Eric R. Burrough^7^, Alejandro Ramirez^7,14^, Shankumar Mooyottu^5,15^, Tanja Opriessnig^7,16^, Ed J. Kuijper^1,2^, Meta Roestenberg^17^, Wiep Klaas Smits ^1,2,*^

^1^ Centre for Microbiome Analyses and Therapeutics, Leiden University Center for Infectious Diseases (LUCID), Leiden University Medical Center, Leiden, The Netherlands

^2^ Experimental Bacteriology, Leiden University Center for Infectious Diseases (LUCID), Leiden University Medical Center, Leiden, The Netherlands

^3^ Leiden Genome Technology Center, Department of Human Genetics, Leiden University Medical Center, Leiden, The Netherlands

^4^ Therapeutic Microbiology Laboratory, Department of Molecular Virology and Microbiology, Alkek Center for Metagenomics and Microbiome Research, Baylor College of Medicine, Houston TX, USA

^5^ Department of Pathobiology, College of Veterinary Medicine, Auburn University, Auburn, AL, USA

^6^ Department of Clinical Sciences, College of Veterinary Medicine, Auburn University, Auburn, AL, USA

^7^ Department of Veterinary Diagnostic and Production Animal Medicine, College of Veterinary Medicine, Iowa State University, Ames, IA, USA

^8^ Department of Biomedical Sciences, College of Veterinary Medicine, Iowa State University, Ames, IA, USA

^9^ Department of Veterinary Biomedical Sciences, Shreiber School of Veterinary Medicine, Rowan University, Glassboro, NJ, USA

^10^ Department of Veterinary Clinical Sciences, College of Veterinary Medicine, Iowa State University, Ames, IA, USA

^11^ Department of Veterinary Medicine and Surgery, College of Veterinary Medicine, University of Missouri, Columbia, MO, USA

^12^ Department of Veterinary Microbiology and Preventive Medicine, College of Veterinary Medicine, Iowa State University, Ames, IA, USA.

^13^ Department of Veterinary Science, Martin-Gatton College of Agriculture, Food and Environment, University of Kentucky, Lexington, KY, USA

^14^ College of Veterinary Medicine, The University of Arizona, Oro Valley, AZ, USA.

^15^ Department of Veterinary Pathology, College of Veterinary Medicine, Iowa State University, Ames, IA, USA

^16^ Department of Vaccines and Diagnostics, the Moredun Research Institute, Edinburgh, UK.

^17^ Controlled Human Infections Center, Leiden University Center for Infectious Diseases, Leiden University Medical Center, Leiden, The Netherlands

**Running title**: Characterization of L-NTCD03

**Key words**: non-toxigenic *C. difficile*, complete genome, antimicrobial resistance, nutrient utilization

* Corresponding author: Experimental Bacteriology group, Leiden University Center for Infectious Diseases, Leiden University Medical Center, PO Box 9600, 2300RC, Leiden, The Netherlands, phone: +31-71-526 1229, fax: +31-71-526 6761, E-mail: [w.k.smits@lumc.nl](mailto:adg@mit.edu)

**Supplementary information**

**Histological scoring**

H&E-stained sections from each ileal loop were graded for four components present in the dataset: epithelial necrosis, edema (mucosal/lamina propria), crypt abscess, and neutrophils in villi/lamina propria. A fifth field, submucosal edema, was also recorded but was invariant (0 in this experiment) and therefore excluded from totals.

Each component used an ordinal 0–5 scale with shared anchors; the best matching description was assigned:

- Epithelial necrosis: 0 = absent; 1–2 = scattered villus-tip necrosis; 3 = multifocal epithelial lifting/patchy denudation; 4–5 = broad denudation/ulceration with diffuse surface loss.
- Edema (mucosal/lamina propria): 0 = none; 1–2 = subtle to mild expansion; 3 = clear expansion with villus blunting; 4 = marked expansion; 5 = diffuse expansion with severe blunting/separation.
- Crypt abscess: 0 = none; 1–2 = rare crypts with a few intraluminal neutrophils/debris; 3 = several crypts with clear pus; 4–5 = numerous/widespread abscesses across fields.
- Neutrophils in villi/lamina propria: 0 = no neutrophils; 1= scattered individual cells; 2 = multifocal small clusters; 3 = multifocal large clusters; 4 = confluent clusters; 5 = sheets/near-diffuse infiltration. (Field counts may aid consistency; final grade is by pattern/severity.)

Total histologic score: per loop, the sum of the four variable components (range 0–20); higher totals indicate greater injury. Each loop was treated as the experimental unit; scoring was performed blinded to group identity.

**Statistical analyses**

*Outcome and unit*. The primary endpoint was the total histologic score per ileal loop (range 0–20), computed as the sum of four 0–5 ordinal components: epithelial necrosis, mucosal/lamina propria edema, crypt abscess, and neutrophils in villi/lamina propria. Submucosal edema values were invariant and excluded from totals. Each loop was the experimental unit; scoring was blinded to group.

*Descriptive statistics*. Group data are summarized as median [Q1–Q3]. Figures display mean ± SEM with per loop jittered points.

*Global comparison*. Overall heterogeneity among Control loop, Normal Ileum, L-NTCD03 and LL-078 was assessed with the Kruskal–Wallis test (two sided, α = 0.05) using mid ranks with tie correction.

*Primary prespecified contrasts*. The biological comparisons of primary interest were LL-078 vs Control loop, LL-078 vs Normal ileum, and LL-078 vs L-NTCD03. Each was tested using a two sided Mann–Whitney U test (Wilcoxon rank sum). Multiplicity was controlled across these three tests using the Holm procedure (family wise α = 0.05). For each contrast we report the Holm adjusted p value and the rank biserial correlation (r) as an effect size.

**Supplementary Table 1. Histopathological analysis of the pig-ileal loop experiments.**

**Supplementary Table 2. Antimicrobial resistance genes of L-NTCD03.**
