# Supplementary material for: Characterization of the clade 4 non-toxigenic C. difficile isolate L-NTCD03 carrying the cfr(B) gene
Source: FEMS Microbes. 2026 Feb 23;7:xtag010. doi: 10.1093/femsmc/xtag010 (PMC12955701; doi:10.1093/femsmc/xtag010)
Supplement: xtag010_Supplemental_Files [file xtag010_supplemental_files.zip › FEMSMC-2026-003.R1 supplementary data - reviews and author response.docx]

**Reviews of manuscript FEMSMC-2026-003**

**Reviewer 1**

**Recommendation: Minor Revision**

This manuscript presents a very thorough and carefully executed genomic and phenotypic characterization of a non-toxigenic Clostridioides difficile strain. From a technical and scientific perspective, the work is of high quality.

However, I have a major concern regarding the authors’ conclusion that strain L-NTCD03 represents a promising candidate for development as a live biotherapeutic product. The strain harbours the cfr(B) gene, which confers resistance to clinically important antimicrobials, including clindamycin and linezolid. Importantly, cfr(B) is a mobile resistance determinant with documented potential for horizontal transfer to other bacterial species.

The deliberate propagation and administration of a strain carrying a transferable resistance gene, particularly one encoding resistance to linezolid, a last-line antibiotic, raises significant safety concerns. The introduction of such a strain into the human gut microbiota could facilitate the dissemination of this resistance determinant.

This limitation should be explicitly acknowledged and discussed in depth. At minimum, the conclusion should be substantially revised to avoid proposing this strain as a therapeutic candidate without addressing the associated antimicrobial resistance risks.

Specific comments

Correct "bioMérieux" throughout the manuscript

Is it necessary to provide again references for individual methods in the result section?

**Reviewer 2: Doğanhan Er, Kocaeli University**

**Recommendation: Minor Revision**

The aim of the current manuscript is to characterize and determine the complete genome sequence of the non-toxigenic *Clostridioides difficile* strain L-NTCD03. The manuscript is cliearly written and requires only minor revisions. The following comments are intended to improve clarity, consistency, and organisation:

-Table headings and their order should be reviewed throughout the manuscript.

-In line 111, “−80 °C” should be added, and in line 113, “37 °C” should be included.

-In line 122, the full name of CDMN should be provided at first mention.

-In line 136, and throughout the manuscript, the term “gradient test” should be used instead of “epsilometer test” or “Etest.”

-In line 137, and throughout the manuscript, “amoxicillin/clavulanic acid” should be used instead of “Augmentin.”

-Throughout the manuscript, more recent CLSI standards should be cited.

In line 148, “Liophilchem” should be corrected.

-Throughout the manuscript, the notation “CO_2_” should be used consistently.

-In line 276, the reference should be checked; the same reference should also be corrected in the References section.

-In lines 291 and 294, there is no need to use the abbreviation “BBA”; please write “Brucella blood agar” in full.

Thank you for the opportunity to review this manuscript. I hope that the above comments will be helpful to the authors in revising and improving their work.

Sincerely,

Reviewer

**Associate Editor Recommendation: Minor Revision**

Thank you for submitting your manuscript to FEMS Microbes. We have now received two external reviews, both of which recommend minor revision.

Both reviewers found the study to be well executed, clearly written, and of high quality, and agreed that the genomic and phenotypic characterization of this non-toxigenic Clostridioides difficile strain represents a valuable contribution to the field.

Reviewer comments largely focused on minor issues of clarity, consistency, and presentation, including terminology, formatting, and reference updates, all of which should be straightforward to address.

One reviewer raised a substantive point regarding the conclusion that strain L-NTCD03 represents a promising candidate for development as a live biotherapeutic product. Specifically, the presence of the cfr(B) antimicrobial resistance gene, which is potentially transferable, raises safety considerations that should be more explicitly acknowledged and discussed. The conclusion should be revised to avoid overstating translational potential without addressing this limitation.

**EIC Decision: Minor Revision**

**Author's Response to Decision Letter for (FEMSMC-2026-003)**

We thank the reviewers for their constructive feedback. We provide a point-by-point response below and additionally provide this response as a PDF for easier review. Our resubmission also includes a version of the manuscript in which the changes have been tracked.

Reviewer: 1

Comments to the Author

This manuscript presents a very thorough and carefully executed genomic and phenotypic characterization of a non-toxigenic Clostridioides difficile strain. From a technical and scientific perspective, the work is of high quality.

However, I have a major concern regarding the authors’ conclusion that strain L-NTCD03 represents a promising candidate for development as a live biotherapeutic product. The strain harbours the cfr(B) gene, which confers resistance to clinically important antimicrobials, including clindamycin and linezolid. Importantly, cfr(B) is a mobile resistance determinant with documented potential for horizontal transfer to other bacterial species.

The deliberate propagation and administration of a strain carrying a transferable resistance gene, particularly one encoding resistance to linezolid, a last-line antibiotic, raises significant safety concerns. The introduction of such a strain into the human gut microbiota could facilitate the dissemination of this resistance determinant.

This limitation should be explicitly acknowledged and discussed in depth. At minimum, the conclusion should be substantially revised to avoid proposing this strain as a therapeutic candidate without addressing the associated antimicrobial resistance risks.

Response

We appreciate the reviewer’s point on the possibility of transferable antimicrobial resistance.

cfr(B) is mainly found in Gram-positive bacteria, such as Enterococci, that are present in the human and animal intestinal tract and are considered as opportunistic pathogens with low virulence. In general, the intestinal tract can be considered as a hidden reservoir for multiple antibiotic resistance genes, including cfr(B) (PMID: 41575223). However, the precise frequency of cfr(B) in other commensal intestinal bacteria and in the human gut resistome is unknown and not systematically studied. Interestingly, a recent study showed high prevalence of cfr(B) in linezolid resistant Enterococci and Lactoccocci cultured from vegetables (PMID: 41250259), suggesting that other sources than C. difficile may be more important.

The presence of cfr(B) gene in methicillin resistant S. aureus (MRSA) is worrying from a clinical perspective, but extremely rare. Horizontal transfer of cfr(B) of C. difficile to MRSA has not been demonstrated and is very unlikely to occur, since MRSA is mainly present in the respiratory tract and skin and not in the intestinal tract. In the Netherlands, it is hypothesized that these (rare) cfr(B)-containing MRSA strains have a livestock background (PMID: 36317053).

In conclusion, we feel that at present there is no evidence for transfer of cfr(B) from this C. difficile strain to potentially pathogenic organisms. Moreover, PhLOPSa antibiotics are not used for treatment of CDI, and the presence of cfr(B) in L-NTCD03 is therefore unlikely to affect CDI treatment.

In the particular case of L-NTCD03, one could even argue that resistance to clindamycin, a major risk factor for the development of CDI, might in fact be beneficial as persistence of L-NTCD03 under clindamycin treatment might protect subjects from developing CDI. Moreover, the presence of reduced susceptibility (PMID 38382090) is no reason to prevent application as a live biotherapeutic products, as is also evidenced from the use of strain NTCD-M3 (vancomycin MIC=2 mg/L, compared to 0.38mg/L for L-NTCD03) in clinical trials (PMID: 25942722). A clinical study, that obtained approval of a medical ethical research committee, has already been conducted with L-NTCD03 (NCTC) underscoring this point (https://clinicaltrials.gov/study/NCT05693077).

To address the reviewer’s concern, however, we have adapted our text in multiple places. The genomic context of cfr(B) is discussed in comparison with other C. difficile strains carrying cfr(B) (L417-422). We acknowledge that at present there is no evidence for transfer, but that this does not exclude the possibility that this can occur (L473-478). We include a suggestion that antimicrobial resistance pattern should be considered before application as a live biotherapeutic product (L493-494). And finally, we indicate that the safety profile of the strain has allowed its use in a clinical trial that was approved by a medical research ethics committee (L494-495).

Specific comments

Correct "bioMérieux" throughout the manuscript

Response

We have corrected the instances of bioMérieux that in our original submission lacked the acute accent.

Is it necessary to provide again references for individual methods in the result section?

Response

We feel that it is appropriate to provide some references to give relevant background to the reader in these sections.

Reviewer: 2

Comments to the Author

The aim of the current manuscript is to characterize and determine the complete genome sequence of the non-toxigenic Clostridioides difficile strain L-NTCD03. The manuscript is cliearly written and requires only minor revisions. The following comments are intended to improve clarity, consistency, and organisation:

-Table headings and their order should be reviewed throughout the manuscript.

Response

We have reviewed the table headings as requested but feel that they are appropriate. As no concrete points for improvement are offered, we kindly refer to the copy editors to further improve the tables, in line with journal style.

-In line 111, “−80 °C” should be added, and in line 113, “37 °C” should be included.

Response

Done.

-In line 122, the full name of CDMN should be provided at first mention.

Response

Done.

-In line 136, and throughout the manuscript, the term “gradient test” should be used instead of “epsilometer test” or “Etest.”

Response

Done.

-In line 137, and throughout the manuscript, “amoxicillin/clavulanic acid” should be used instead of “Augmentin.”

Response

Done.

-Throughout the manuscript, more recent CLSI standards should be cited.

Response

We appreciate this feedback. However, when experiments were originally conducted, these were the CLSI standards used by our lab. We feel it is inappropriate to refer to newer documents that were not actually used at the time of testing. This does not affect the conclusions in the manuscript.

In line 148, “Liophilchem” should be corrected.

Response

Corrected to Liofilchem.

-Throughout the manuscript, the notation “CO2” should be used consistently.

Done.

-In line 276, the reference should be checked; the same reference should also be corrected in the References section.

Response

We thank the reviewer for pointing out the apparent error in this reference, caused by the automated formatting of the reference software. We have corrected this to (ECDC, 2018) in the text, and adapted the reference in the Reference list.

-In lines 291 and 294, there is no need to use the abbreviation “BBA”; please write “Brucella blood agar” in full.

Response

Done.

**Reviews of manuscript FEMSMC-2026-003.R1**

**Associate Editor Recommendation: Accept**

Thank you for your careful and thorough revision and for the detailed, point-by-point responses to the reviewers’ comments. After evaluation of the revised manuscript and responses, I am pleased to inform you that the manuscript has been accepted for publication.

The reviewers’ concerns—particularly regarding antimicrobial resistance considerations—have been appropriately addressed through revisions to the text and clarifications provided.

**EIC Decision: Accept**
